# Folate deficiency reduced aberrant level of DOT1L-mediated histone H3K79 methylation causes disruptive SHH gene expression involved in neural tube defects

**DOI:** 10.1186/s13072-023-00517-3

**Published:** 2023-12-14

**Authors:** Xue Li, Pei Pei, Jinying Shen, Juan Yu, Fang Wang, Lei Wang, Changyun Liu, Shan Wang

**Affiliations:** 1 Weifang People’s Hospital, Weifang, 261041 Shandong China; 2 School of Clinical Medical, Weifang Medical University, Weifang, 261053 Shandong China; 3 Beijing Municipal Key Laboratory of Child Development and Nutriomics, Capital Institute of Pediatrics, Beijing, 100020 China; 4 Department of Basic Medical Sciences, Changzhi Medical College, Changzhi, 046000 China; 5 Institute of Basic Medical Sciences, Chinese Academy of Medical Science, Beijing, 100730 China

**Keywords:** Histone methylation, Neural tube defects (NTDs), Methotrexate (MTX), Disruptor of telomeric silencing 1-like (DOT1L), Sonic hedgehog (SHH)-related genes

## Abstract

**Background:**

Neural tube defects (NTDs) are one of the most severe congenital abnormalities characterized by failures of the neural tube to close during early embryogenesis. Maternal folate deficiency could impact the occurrence of NTDs, however, the mechanisms involved in the cause of NTDs are poorly defined.

**Results:**

Here, we report that histone H3 methyltransferase disruptor of telomeric silencing 1-like (DOT1L) expression was significantly downregulated, and low levels of H3K79me2 were found in the corresponding NTDs samples with their maternal serum folate under low levels. Using ChIP-seq assays, we found that a decrease of H3K79me2 downregulates the expression of *Shh* and *Sufu* in mouse embryonic stem cells (mESC) under folate deficiency. Interestingly, folate antagonist methotrexate treatment led to attenuation of H3K79me2 due to Dot1l, affecting *Shh* and *Sufu* genes regulation. Upon further analysis, we find that the genes Shh and Sufu are both downregulated in the brain tissues of mice and humans with NTDs. There was a positive correlation between the transcription levels of *Shh*, *Sufu* and the protein levels of DOT1L by Pearson correlation analysis.

**Conclusion:**

Our results indicate that abnormal *Shh* and *Sufu* genes expression reduced by aberrant Dot1l-mediated H3K79me2 levels could be the cause of NTDs occurrence.

**Supplementary Information:**

The online version contains supplementary material available at 10.1186/s13072-023-00517-3.

## Introduction

Neural tube defects (NTDs) are severe congenital malformations that result from failure of neural plate patterning and tissue fusion. Failure of the completely closure in craniofacial region causes exencephaly, and if the exposed brain tissue is degraded, it appears as anencephaly, which usually causes embryonic lethality [1–3]. Research has indicated that gene–gene, gene–environment, and maternal genetic alteration could be NTDs risk factors [4]. Folic acid supplementation has greatly reduced the incidence of folate-dependent NTDs during periconceptional period [5, 6]. Folate-mediated one carbon metabolism is associated with NTDs [7]. Studies have found that the biosynthesis of thymidine and purine is abnormal in NTDs mouse models [8–10]. The lack of folic acid plays a certain role in the occurrence of NTDs, but the specific mechanism of NTDs in human is still unclear [11, 12].

Studies have shown that dysregulation of gene expression through epigenetic mechanisms can potentially result in formation of NTDs [13]. Mouse models have indicated that aberrant histone methylation may lead to exencephaly. Methylation on various H3 lysine positions, such as H3K4me1, H3K27me3, H3K36me3 and H3K79me2 show that distinct chromatin patterns are involved in the stage-specific gene expression during embryonic development and temporal alterations of programmed histone modification distinguish the key genes regulation in NTDs [14, 15]. Elevated homocysteine (Hcy) upregulates H3K79Hcy expression and promotes NTDs resulting in an abnormal expression for selected neural tube closure(NTC)-related genes [17]. Our previous study indicated that folate deficiency attenuated H3K79me2, affected its activation in target genes. These mechanisms were known to be associated with NTDs, and interrupt early embryo development [18]. However, the molecular mechanism underlying the connection between folate deficiency, histone methylation and the occurrence of NTDs remains unclear.

Histone methyltransferase is associated with transcriptional regulation, and particularly important to neural tube closure. In our earlier reports, we found that low folate-induced H3K79me2 promoted gene activation, some of which were known to be associated with NTDs and early embryonic development [18]. H3K79me2 occurs primarily at the promoters of actively transcribed genes. DOT1L (disruptor of telomeric silencing 1-like, also known as KMT4) is an evolutionarily conserved histone methyltransferase that catalyzes the monomethylation, dimethylation, and trimethylation of histone H3 at lysine79 [19]. Dot1L is essential for embryonic development, hematopoietic function and cardiac function. The methylation of H3K79 is catalyzed by the methyltransferase DOT1L, and it requires the assistance of the H2BK120ub1. Recent structural studies have revealed the mechanism by which H2BK120ub1 activates Dot1L enzymatic activity. The ubiquitin group on H2BK120 and the acidic patch of H2A/H2B jointly localize and stabilize DOT1L on nucleosome. Then, the catalytic active site of DOT1L was relocated just above the H3K79 residue while the interaction between H4 tail and DOT1L happened. As a result, the conformation changes occurring at the H3K79 residue localize itself to the DOT1L catalytic active site, completing the methylation reaction [20]. DOT1L-mediated H3K79 methylation has an effect on several processes, such as embryonic development, erythropoiesis, cardiac differentiation and certain cancers. Mutation in DOT1L resulted in abnormal H3K79 methylation and downstream gene dysregulation [21]. Results demonstrate that histone methylation, and in particular DOT1L-mediated H3K79me2 modification, drives cardiomyogenesis through the definition of a specific transcriptional landscape [22]. DOT1L activity not only plays important roles on CNS development in the cerebral cortex and cerebellum, but also keeps balances on progenitor proliferation and differentiation in other somatic organ systems [23–26].

In mouse and human, more than 200 genes are found to play key roles in the formation of NTDs. Human are multifactorial, polygenic and more complicated than other animals [27–29]. However, genetic polymorphisms only led to certain types of NTDs and some of the NTDs-related genes identified in mice are also involved in human. On various occasions, because of the complexity and dynamics of NTC, the relationship between gene mutations and NTDs remains unclear. In summary, reports showed several major pathways involved in NTDs are PCP pathways, SHH pathways, Wnt–Catenin pathways, Notch pathway and PI3K–AKT pathways. In early forebrain and central nervous system development, the SHH signaling pathway is involved in the regulation of the dorsal–ventral formation, proliferation and differentiation of neural precursor cells in multiple regions. This pathway plays a key role in neural progenitor cell growth [30]. In the study of vertebrate, mutation of core genes, such as *Shh*, *Ptch1*, *Smo*, *Cdon*, *Sufu* and *Gli* in Shh signaling pathway lead to the occurrence of NTDs or abnormal embryonic development. In maintaining normal operation of the SHH signaling pathway, the distribution and expression of these genes play a vital role. In human embryo, *SHH* is expressed in the notochord, the floorplate, the brain, and *SHH* mutations caused holoprosencephaly [31]. In Shh-knockout mice, ventral cells and floor plate formation are defective [32] and holoprosencephaly and cyclops develop because of failure in cranial neural tube closure. *Sufu* gene is located at 10q24.32 and is an important negative regulator of SHH signaling pathway. In cranial NTDs, single-site or multi-sites spina bifida were manifested when *Sufu* mutations happen [33].Current research found that Dan showed expression in cranial mesenchyme and somites at E8.5, then in limb and facial mesenchyme during embryogenesis [34]. Our previous study found that a decreased of H3K79me2 binds to *SUFU* gene in NTDs brain tissues. The results showed loss of H3K79me2 occurred in some NTDs [18]. However, the molecular mechanism that DOT1L-mediated H3K79me2 on SHH pathway regulation in occurrence of folate-deficiency-induced NTDs are still unknown.

Our study found that DOT1L was significantly downregulated, and low levels of H3K79me2 were found in the corresponding NTDs samples with their maternal serum folate under low folate levels. Using ChIP-seq assays, we found that a decrease in H3K79me2 downregulates the expression of *Shh* in mouse embryonic stem cells (mESC) under folate antagonist methotrexate. Interestingly, folate deficiency led to attenuation of H3K79me2 due to Dot1l, affecting *Shh* and *Sufu* genes regulation. On further analysis, downregulation of *Shh* and *Sufu* genes was also found in brain tissues of mouse and human cases. There was a positive correlation between transcription levels of *SHH*, *SUFU* and protein levels of DOT1L by Pearson correlation analysis. Together, our results provide evidence that folate deficiency affects DOT1L activity and H3K79me2 levels, resulting in the abnormal expression of Shh, Sufu genes and subsequently NTDs. It extends our understanding of aberrant epigenetic modification of NTC-related genes in NTDs.

## Materials and methods

### Ethics and participants

All clinical samples were collected from Lvliang in Shanxi province in China with signed informed consents from patients and their families. The protocol was approved by the Ethics Board of Capital Institute of Pediatrics. All animal experiments were legally performed under "Principles for Utilization and Care of Vertebrate Animals".

### Nanostring

NanoString is a high-throughput method for RNA expression detection. The total RNA of human brain tissue was extracted and detected by NanoString nCounter. First, gene-specific probes were designed. Sample lysate was then incubated and hybridized with the probes. The probes were designed by NanoString Technologies and hybridized according to the nCounter Element 24-plex Assay Manual. The hybridization solution was incubated at 65 ºC for 20 h. The remaining hybridization mixture was added to Cartrige through a series of hybridization and elution processes, and then the cartrige was placed on an optical scanner. Data were filtered using quality control (QC) criteria according to the manufacturer's recommendations. GAPDH, CLTC, and GUSB were used as internal controls to normalize the raw counts to pass QC. The data used for analysis were transformed by log_2_.

### Animals

C57BL/6 mice (6–8 weeks, 18–20 g) were purchased from Charles River Laboratory, and fed in a specific pathogen-free environment under a 12-h light/dark cycle. Female C57BL/6 mice were fed on low-folate diet for 4 weeks. Sexually matured individuals were mated overnight in the morning at 8:00 am. This time was seen as embryonic day (E) 0.5. On E7.5, mice were intraperitoneally injected with 1.5 mg/per kg of MTX (Sigma, USA). Pregnant mice were killed, and the dissected embryos were stored in liquid nitrogen. Animal handling was compliant with institutional guidelines under the care of experimental animals.

### Cell culture

Sv/129 mouse embryonic stem cells (mESCs) were obtained from Capital Institute of Pediatrics central laboratory and cultured in 15% fetal bovine serum (FBS), DMEM (Gibco), non-essential amino acids (Invitrogen), 0.1 mM glutamine (Invitrogen), and 1,000 U/ml mouse leukemia inhibitory factor(LIF) (Millipore, Billerica, USA). Cells were cultured in 0.2% gelatin-coated T25 bottles and incubated in an incubator at 37 ºC 5% CO2, and the medium was changed every day. Cells were cultured under normal medium for few days before 1uM methotrexate (MTX) was applied for 24 h.

### Western blotting

Histone used in this paper were all obtained by acid extraction. We used 12% SDS-PAGE to isolate 5ug of histones and detected the changes in the target protein. Protein was transferred to an immobilon-NC membrane (Merck Millipore, Ireland) by electroporation, and the membranes were incubated with the primary antibody, anti-Histone H3 (di methyl K79) antibody (1: 1000, abcam, Cambridge, UK), DOT1L (D1W4Z) Rabbit mAb (1: 1000, Cell Signaling Technology, USA), and Anti-Histone H3 antibody (1: 5000, Abcam, Cambridge, UK) and stored under four-degree refrigerator overnight. Membranes were washed with PBST for three times on the next day and incubated in secondary Peroxidase-conjugated goat anti-rabbit IgG antibody (1:5000, ZSGB-BIO, China) for 40 min.

### ChIP-Seq and data analysis

10^7^ mESC cells were used to obtain DNA with a concentration of 1 ng/ul and fragments were digested into100-500 bp for CHIP(chromatin immunoprecipitation) sequencing. SimpleChip KIT (Cell Signaling Technology, America). CHIP-Seq libraries were prepared according to the protocol and sequenced using Illumina NovaSeq 6000. After passing the quality check, all reads are mapped to mouse genome using Bowtie2 with default parameters. Two mismatches cannot appear in one cohort. The peak of the H3K79me2 on the genome was determined using MACS2 with a false detection rate of 0.05. Each peak-associated gene was identified by an internal program using Entrez gene annotation, where the Shh target gene was defined as a gene containing H3K79me2-peak in its transcription start site (TSS). The data set of H3K79me2 reads per kilobase per million (RPKM) values in the coding region of each gene was normalized.

### Chromatin immunoprecipitation (ChIP) analysis

SimpleChip KIT (Cell Signaling Technology, America) was used. Chromatins were ultrasonicated for 100–500 bp DNA fragmentations, then immunoprecipitated with H3K79me2 XP Rabbit mouse antibody (CST, USA). The purified DNA was detected by QuantStudio 7 Flex with SYBR Green by RT-qPCR. The primers used for RT-qPCR are shown in Additional file 1: Table S1. Normal Rabbit IgG was used as a negative control.

### RT-qPCR

Trizol was used to extract mRNA from mouse cranial neural tissues and cells. We used the RevertAid First Strand cDNA Synthesis Kit (Thermo, USA) to synthesize the single strand cDNA, followed by Maxima SYBR Green / ROX qPCR Master Mix (Thermo, USA) for subsequent qPCR experiments. The qPCR setup procedure is as follows: cDNA was initially denatured at 95 ºC for 3 min with 1 cycle, then held for 30 s, Annealing temperature was raised to 50 ºC for 30 s and elongated at 72 ºC, for 1 min, number of cycles from denaturation to extension was 40. Finial extension lasted for 5 min at 72 ºC. Primer sequences are shown in Additional file 1: Table S1.

### Small interfering RNA (siRNA)

siRNA negative control, AUGUAUUGGCCUGUAUUAG.

DOT1L siRNA, GCUAUGGAGAAUUACGUUU.

#### Immunofluorescence

Cells were fixed in 4% paraformaldehyde and placed at room temperature for 10 min, then 1 ml of 0.1% Triton X-100 in PBS was added and shook on a shaker for 5 min. Cells were then blocked with 5% BSA blocking solution for 1 h at room temperature. Cells was incubated with primary antibodies DOT1L and H3K79me2 (1:50; CST) (1:50; Santa Cruz) and placed in a 4-degree refrigerator overnight. Followed by incubation with Alexa Fluor-conjugated secondary antibodies (1: 200; CST, USA) for 1 h at 37° C. DAPI staining was then added for 10 min. Images were observed under a Leica laser scanning confocal microscope. Colocalization values of DOT1L and H3K79me2 were calculated using FV10-ASW 3.0 Viewer.

#### Statistical analysis

All statistical analyses were performed with SPSS software, version 19.0. Student-t test was used to calculate means and standard deviation. Statistically significant is when p-value is under 0.05.

## Results

### Decreased DOT1L expression in low-folate NTDs fetuses

**Fig. 1:**
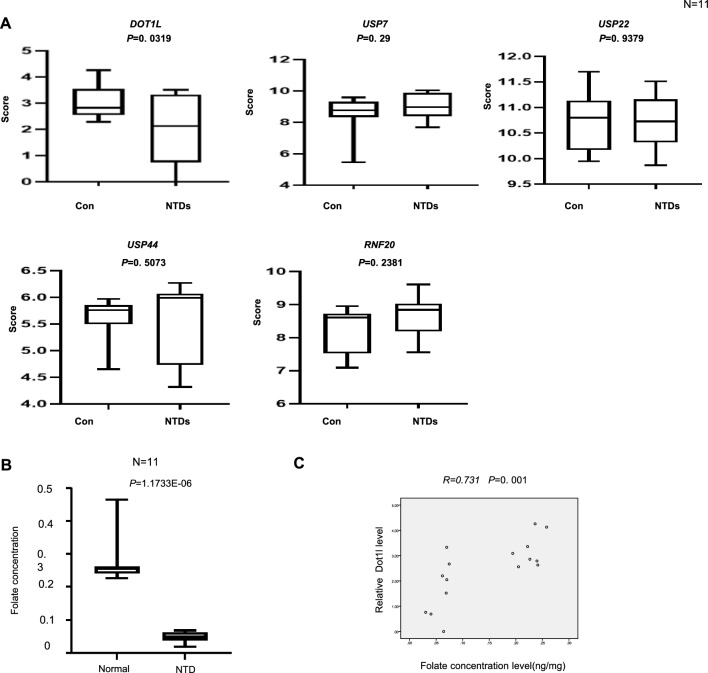
Decreased expression of *DOT1L* gene in low-folate human NTDs fetuses. **A** The mRNA level of DOT1L, USP7, USP22, USP44, RNF20 was detected by Nanostring in the NTDs fetuses (n = 11). **B** Folate concentration of normal fetuses and NTDs fetuses was shown as box plots. **C** Pearson’s correlation analysis was used between the expressions of DOT1L genes and folate level. All data are shown in mean ± sd. (n = 11), *p > 0.05

**Fig. 2:**
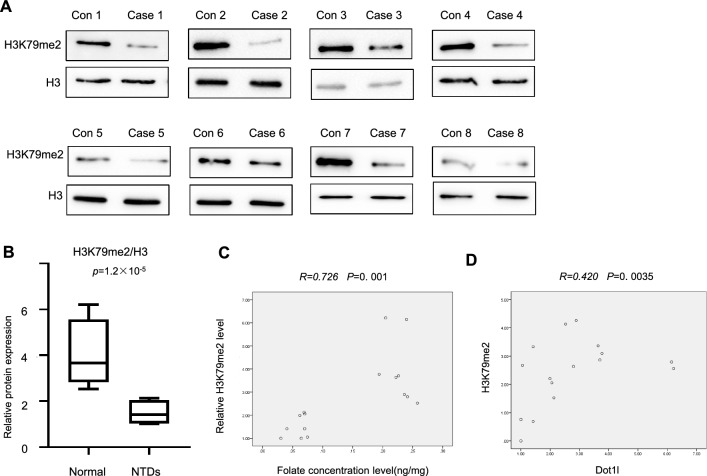
Aberrant H3K79me2 expression in low-folate human NTDs fetuses. **A** Changes of H3K79me2 in brain and spinal tissues of human NTDs fetuses and normal fetuses were detected by western bloting, H3 was used as a loading control. **B** Box-plot was used to quantify signal intensity of H3K79me2 western blot. (Sample size in Normal and NTDs groups, respectively: n = 8.) **C** Pearson’s correlation analysis was shown between H3K79me2 protein expression of human sample and their maternal folate level. All above data are mean ± sd. (n = 11), *p < 0.05. **D** Pearson’s correlation analysis was shown between the transcriptional expressions of *DOT1L* and H3K79me2 protein levels in human samples

The failure of neural tube closure led to occurrence of NTDs, which is a serious deformity that has severe consequences to individuals. The methylation of H3K79 is catalyzed by the methyltransferase DOT1L, but it requires the assistance of the H2BK120ub1. To explore the potential role of H3K79me2 and H2BK120ub1 in NTDs, we evaluated the expression of DOT1L, enzymes involved in H2BK120 monoubiquitination and deubiquitination USP7, USP22, USP44, RNF20 in brain of normal fetuses and low-folate related NTDs via NanoString assay. 11 pairs of the human fetuses with spina bifida were selected as summarized in Additional file 2: Table S2. The mRNA expression of *DOT1L* was significantly downregulated in NTDs tissues when compared with normal tissues (*p* < 0.05; Fig. 1A). No significant difference in expression was found in *USP7*, *USP22*, *USP44*, *RNF20*. We evaluated brain tissues of the NTDs fetuses and their maternal serum folate levels. The maternal serum folate level was significantly lower compared with control (Fig. 1B).Importantly, levels of *DOT1L* was correlated with maternal serum folate levels. These results suggested that DOTL1 is associated with low-folate NTDs. Western blot analysis indicated that H3K79me2 levels were decreased in eight NTDs samples compared with normal (Fig. 2A). The average levels of H3K79me2 (normalized to H3) were sharply decreased in NTDs samples (*p* < 0.05; Fig. 2B). Interestingly, levels of H3K79me2 was also correlated with maternal serum folate (Fig. 2C). We also found a significant positive correlation between H3K79me2 and *DOT1L* (Fig. 2D).

### MTX reduced enrichment of H3K79me2 in Shh and SuFu gene promotors

**Fig. 3:**
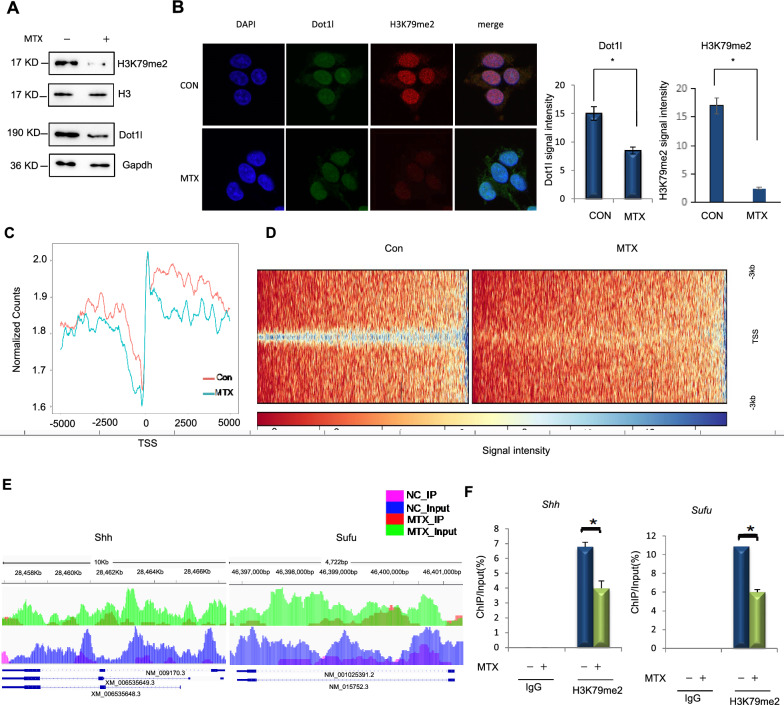
MTX reduced enrichment of H3K79me2 in *Shh* and *Sufu* genes promoters. **A** Mouse ESCs were treated with MTX (1 μM) for 12 h. Protein was extracted and subjected to western blotting and detected by Dot1L and H3K79me2 antibodies. **B** F9 cells were treated with MTX (1 μM) for 12 h for immunofluorescence detection. **C**, **D** ChIP-Seq H3K79me2 assemble around TSSs. ChIP-Seq: enrichment of H3K79me2 in mESC cells under MTX (1 μM) 12 h. **E** ChIP-seq: enrichment of H3K79me2 in *Shh*, *Sufu* genes in MTX-treated mESC. **F** ChIP assays were performed using H3K79me2 antibody in MTX-treated mESCs (1 μM, 12 h). Mouse IgG was used as negative control. Enrichment of H3K79me2 in *Shh*, *Sufu* genes were detected by RT-qPCR. Data are shown as mean (n = 3). *p < 0.05, by Student’s t-test

Folate antagonist MTX, as an inhibitor of dihydrofolate reductase, prevents the conversion of folate to its active form and antagonizes the function of folic acid metabolism. NTDs formed in later generation when low folate-fed maternal mice were under MTX treatment. Preliminary studies in our laboratory found that the incidence of NTDs mouse embryos was significantly increased when their mother under low-folate diet for at least 4 weeks. Our previous study showed that H3K79me2 was reduced in folate-deficient mouse embryonic stem cells (mESC) [17, 18]. We detected a reduction of H3K79me2 and Dot1l in MTX-treated mESC (Fig. 3A). H3K79me2 and Dot1l were significantly colocalized to nuclear foci under MTX-treated mESC cells (Fig. 3B). ChIP-seq of H3K79me2 showed that the enrichment level of H3K79me2 was decreased in MTX-treated mESC compared to controls (Fig. 3C). To learn more about the role of H3K79me2 during embryonic development, we analyzed equal number of reads and statistical enrichment of H3K79me2 and target genes (Additional file 3: Tables S3). ChIP in GO and KEGG showed that H3K79me2 target genes distributed in different pathways (Additional file 4: Tables S4 and Additional file 5: Table S5) and identified a remarkable reduction of H3K79me2 near transcription start sites in MTX-treated mESC (Fig. 3D). Next, we analyzed the specific region and accumulation of H3K79me2 peaks in *Shh* and *Sufu* genes. The result showed that accumulation of H3K79me2 in those genes was reduced (Fig. 3E). Besides, chromatin immunoprecipitation (ChIP) assays were performed to evaluate the enrichment of H3K79me2. As shown in Fig. 3F, enrichment of H3K79me2 in *Shh* and *Sufu* genes were attenuated. By contrast, no change in enrichment of H3K27me3 in IgG loci was observed (Fig. 3F). Collectively, our data indicated that the enrichment of H3K79me2 on *Shh* and *Sufu* genes were decreased, their transcription levels were increased.

### MTX-induced downregulation of *Shh* and* Sufu* genes expression depends on Dot1l-mediated reduction in H3K79me2

**Fig. 4:**
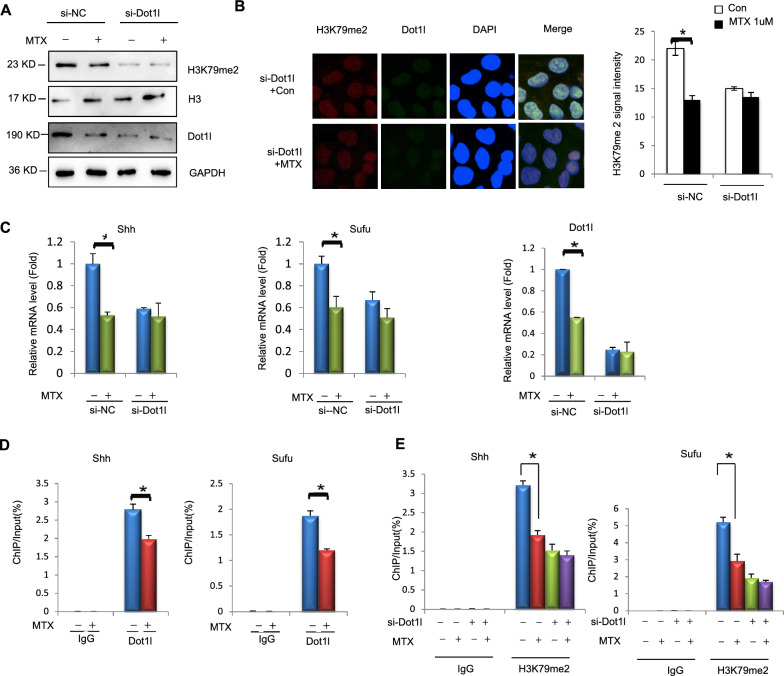
MTX-induced downregulation of *Shh*, *Sufu* genes expression depends on Dot1l. **A** siRNA-*Dot1l* and siRNA-Con were transfected for 24 h in (mouse teratocarcinoma cells) F9 cells, and then cells were treated with MTX for 12 h. Protein was extracted for Western blotting. **B**, **C** F9 cells were transfected with siRNA-*Dot1l* for 24 h, and then treated with MTX (1 μM, 12 h) for immunofluorescence and RT-qPCR detection. **D** Chromatin immunoprecipitations were performed using digested chromatin from MTX (1 μM, 12 h) treated mESCs and 77,087 Dot1L(D1W4Z)Rabbit mAb. The enrichment of Dot1L at *Shh*, *Sufu* genes promoters by RT-qPCR. **E** F9 cells siRNA-*Dot1l* and con were transfected for 24 h and treated with MTX for 12 h. Chromatin immunoprecipitations were performed using digested chromatin from F9 cells and H3K79me2, and the enrichment of H3K79me2 at *Shh*, *Sufu* genes promoters or intron was detected by RT-qPCR

Our previous analyses demonstrated that enrichment of H3K79me2 in *Shh* and *Sufu*, was significantly decreased in NTDs brain tissues [18]. We studied the effects of *Dot1l* depletion on H3K79me2. We detected the H3K79me2 in *Dot1l* depletion-F9 cells (mouse embryonic carcinoma cell). MTX induction decreased H3K79me2, and *Dot1l* depletion released MTX induction on H3K79me2 (Fig. 4A). *Dot1l* depletion attenuated MTX induction on H3K79me2 (Fig. 4B). Downregulation of *Shh*, *Sufu* and *Dot1l* was attenuated after *Dot1l* knockdown in F9 (Fig. 4C). Furthermore, enrichment of Dot1l to promoters transcriptionally activated *Shh* and *Sufu* (Fig. 4D and C). In folate-deficient mESCs, enrichment of Dot1l in gene promoter regions transcriptional activated *Shh* and *Sufu* genes and IgG showed slight enrichment without significant changes. (Fig. 4D). We detected that the depletion of Dot1l attenuated enrichment of H3K79me2 in *Shh* and *Sufu* genes (Fig. 4E). In conclusion, above results demonstrated that the downregulation of Dot1l interfered with H3K79me2 had effect on *Shh* and *Sufu* in F9 cells, indicating that Dot1l is required for transcriptional expression of *Shh* and *Sufu* genes (Additional file 1).

### Supplementation with folinic acid increases H3K79me2 induced by MTX and affects enrichment of H3K79me2 in *Shh* and *Sufu* genes

**Fig. 5:**
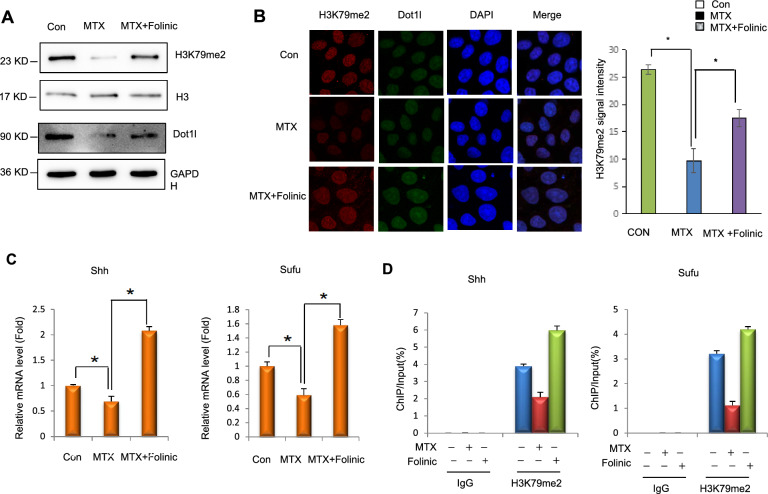
Folinic acid attenuates MTX-reduced enrichment of H3K79me2 in *Shh*, *Sufu* genes. **A** Western blot assay analyzed as indicated antibody. mESCs were cultured in complete medium under MTX treatment (1 μM; 12 h), and supplemented with folinic acid (50 mg/L; 24 h). **B** Immunofluorescence was performed on indicated antibodies H3K79me2 and Dot1L in mESCs under MTX (1 μM; 12 h) treatment and folinic acid supplementation. **C** mESCs cells were treated with MTX and replenishing with folic acid, and the mRNA levels of Shh-related genes *Shh*, *Sufu* was detected in RT-qPCR. All data were obtained in three repeated experiments (n = 3). *p < 0.05. **D** mESCs were treated with MTX and supplemented with folic acid, ChIP experiments of H3K79me2 were performed, and the enrichment of H3K79me2 in *Shh*, *Sufu* genes were observed by ChIP-qPCR. All data were obtained in three repeated experiments (n = 3), *p < 0.05

Previously we found that the folinic acid supplementation has influenced upper binding factor (UBF) enrichment on DNA breakage sites in rRNA genes [16]. Studies found that the folinic acid supplementation has decreased MTX-induced toxicity [35]. However, whether folinic acid affects the change of MTX-induced H3K79me2 and mESC differentiation is unknown. In this study, folinic acid (50 mg/L) was used in mESCs culture medium. The H3K79me2 level was elevated and rescued after folinic acid supplementation (Fig. 5A). Dot1l and H3K79me2 foci were significantly reduced in MTX and increased in folinic acid supplementation (Fig. 5B). Next, the expression of *Shh* and *Sufu* was decreased in MTX and increased in folinic acid supplementation (Fig. 5C). We next detected the enrichment of H3K79me2 in and Shh and Sufu promoters in ChIP-qPCR. After folinic acid supplementation, enrichment of H3K79me2 in these two genes were markedly increased but MTX treatment led to a reduction (Fig. 5D). In conclusion, folinic acid supplementation reversed enrichment of H3K79me2 in *Shh* and *Sufu.*

### Low folate reduced the levels of Dot1l and H3K79me2 in NTDs mice

**Fig. 6:**
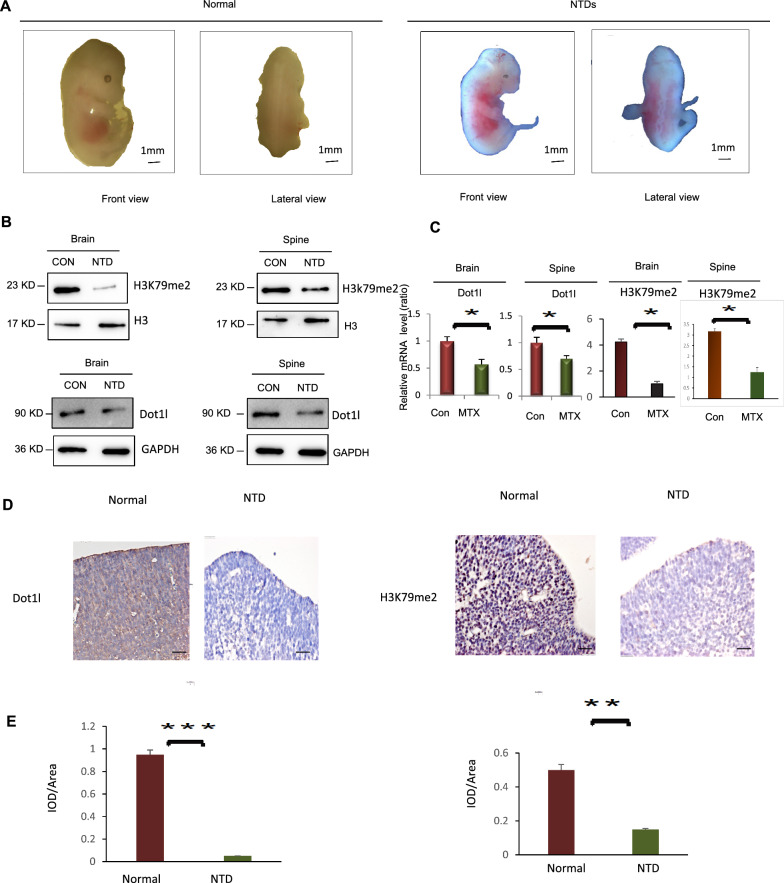
MTX reduced levels of Dot1l and H3K79me2 in NTDs mice. **A** Low folate feed and MTX injection induced NTDs in C57BL/6 mouse embryos at 13.5 days. **B** The brain and spinal tissues of mouse embryos induced by MTX were obtained, collect proteins from the tissues were extracted, Dot1l and H3K79me2 detection by western blot. **C** RT-qPCR was used to detect the mRNA levels of *Dot1l* in brain and spinal tissues of NTDs mice. Data are mean ± s.d. (n = 3). *p < 0.05. **D** Representative images from immunohistochemical staining of H3K79me2 in the cranial neural tissue from E10.5. **E** H3K79me2 expression scores are shown as box plots, with the horizontal lines representing the median. Data are mean ± s.d. (n = 3), *p < 0.05, by Student’s t test

MTX-treated mice under folate deficiency gave birth to offspring with NTDs [36]. We have previously established NTDs mouse models in vivo with low-folate diet and MTX injection. MTX treatment (1.5 mg/kg) is given at E7.5 (equivalent to the 22nd-30th days of human pregnancy), and the morphological changes were prominent at E13.5. We observed a completely closed neural tube in normal mouse and a failed closure at dorsal axis leading to spina bifida under low-folate diet with MTX-injected mouse (Fig. 6A). Folate deficiency during early pregnancy resulted in failure in neurogenesis. Western blot analysis of relative histone methylation demonstrated that there were lower protein levels of H3K79me2 in MTX-induced NTDs samples than normal (Fig. 6B). H3K79me2 was markedly decreased in defected spine at E13.5, suggesting aberrant H3K79me2 resulted in NTDs in mouse. A decreased Dot1l was also observed in MTX-treated NTDs (Fig. 6B). We next examined H3K79me2 level in cranial neural tissue samples and matched normal tissues by IHC analysis. The staining of total H3K79me2 was decreased in mouse NTDs compared with normal tissues (Fig. 6D, E).

### Aberrant expression of* Shh *and *Sufu* genes in low-folate human NTDs fetuses

**Fig. 7:**
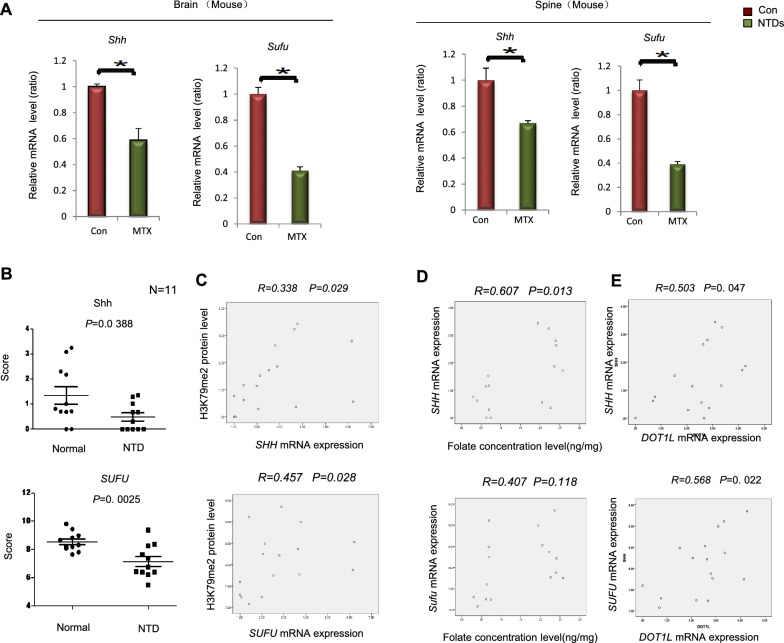
Decreased expression of *SHH*, *SUFU* genes both in low-folate mouse and human NTDs fetuses. **A** RT-qPCR was used to detect the mRNA levels of *Shh*, *Sufu* genes in brain and spinal tissues of NTDs mice. Data are mean ± s.d. (n = 3). *p < 0.05. **B** Nanostring assay was used to detect the mRNA level of *SHH*,*SUFU* genes in the NTDs fetuses’ brain. **C** Pearson’s correlation analysis was shown between H3K79me2 expression and *SHH*, *SUFU* genes. **D** Pearson’s correlation analysis was shown between folate level and *SHH*, *SUFU* genes. **E** Pearson’s correlation analysis was displayed between DOT1L expression and *SHH*, *SUFU* genes. All above data are mean ± sd. (n = 11), *p < 0.05

Because the SHH signaling pathway played an important role in the formation of neural tube defects, we then evaluated the changes in the expression of Shh and Sufu genes at E13.5. The mRNA levels of *Shh* and *Sufu* were significantly decreased in NTDs mouse embryos compared with controls (Fig. 7A; *p* < 0.05). Next, we examined the expression of *SHH* and *SUFU* in low-folate NTDs fetuses. The expression of *SHH* and *SUFU*, were downregulated in human NTDs fetuses (Fig. 7B; *p* < 0.05). We also found a significant positive correlation between H3K79me2 and *SHH*, *SUFU* by correlation analysis (Fig. 7C; *p* < 0.05). In low-folate deficient human NTDs samples, H3K79me2 protein levels were downregulated and positively correlated with the expression of *SHH*, *SUFU*, which was consistent with the results from the NTDs mouse model, indicating that H3K79me2 functions in the early embryonic neurodevelopment through interactions with the *SHH* and *SUFU*. Altogether, these findings demonstrated that the decrease of DOT1L-mediated H3K79me2 was accompanied with a reduction of *SHH*, *SUFU* in low-folate NTDs.

## Discussion

NTDs are the most common congenital malformations in humans resulting from defects in the development of the central nervous system. However, the specific mechanism and the etiology of NTDs in humans are still unknown [37]. Maternal folate levels are important dietary factors related to NTC, the study demonstrated that supplementation with 4 mg folic acid per day resulted in a threefold reduction in NTDs occurrence [38]. In our study, we found that DOT1L expression was significantly downregulated, and low levels of H3K79me2 were found in the corresponding NTDs samples with their maternal serum folate under low levels. Furthermore, folate deficiency led to attenuation of H3K79me2 due to Dot1l, affecting *Shh* and *Sufu* genes regulation. Furthermore, downregulation of Shh and Sufu genes was also found brain tissue of both mouse and human NTDs cases. There was a positive correlation between transcription levels of *SHH*, *SUFU* and protein levels of DOT1L by Pearson correlation analysis. Together, our results provide evidence that folate deficiency affects DOT1L activity and the levels of H3K79me2, which is related to abnormal expression of SHH, SUFU genes and subsequently NTDs. More Studies are required to show the function of folate on neural tube malformation from epigenome and transcriptome. Folic acid is an oxidizing nutrient that affects the production of phenotypes through epigenetics and plays an important role in the epigenetic programming and has a major impact on health and disease, folate is critical for the supply of methyl groups and enzymatically reduction to tetrahydrofolate in cells [39]. In this study, we showed an effect of folate on H3K79me2. Recent studies showed that folate deficiency affects DNA breakage through histone H3K4me1 [16]. Our data found that H3K79me2 was decreased in NTDs neural tissues. In mouse and human NTDs model, the decreased H3K79me2 is associated with open spina bifida (Figs. 2A and 6B). Epigenetic effects are mediated principally by DNA and histone methylation. Our results showed that H3K79me2 was decreased under folate deficiency, but increased after folinic acid supplementation (Fig. 3A). Folate deficiency resulted in reduced histone methylation; conversely, high folate status led to increased histone methylation and, hence, folate status determines epigenetic events. A recent study on folate supplementation and deficiency in human subjects at risk of NTDs reported significant effects of folate on the regulation of gene expression. The loss of histone methylation leads to dysregulated gene expression, which may contribute to NTDs. Folate levels affect gene expression via histone methylation. In this study, we found that decreased H3K79me2 altered the expression of *SHH*, *SUFU* genes in folate-deficient NTDs samples (Fig. 7C). Evidence for this concept has emerged recently in a report on the effects of folate on histone methylation at *Hes1* and *Neurog2* gene loci [40]. In mouse embryos with *Pax3*-deficiency, histone H3K27 dimethylation, a repressive epigenomic mark, is increased at the promoter regions, whereas treatment of folate induced reduction of methylation to its normal levels [41]. This result demonstrates that folate can modulate epigenetic chromatin marks.

Epigenetic transferase involved in neural tube in a temporally and spatially specific manner during development. DOT1L is the methyltransferase of H3K79me2. We investigated the role of *Dot1l* in mESCs under folate deficiency. In *Dot1l* depleted mESC, folate deficiency had no effect on H3K79me2 (Fig. 4A). DOT1L catalyzes the formation of H3K79me1, H3K79me2 and H3K79me3 [42]. Previous reports have proposed a role of DOT1L in development. DOT1L deficiency impairs granule cell proliferation and neuronal differentiation during cerebellar development. However, the role of DOT1L-mediated H3K79me2 modification in the neural tube requires further study. We found that knockdown of *Dot1l* interfered with MTX-reduced H3K79me2 on *Shh*, *Sufu* genes, suggesting that *Dot1l* is required for the expression of those genes (Fig. 4). Our findings indicate the importance of DOT1L in the epigenetic regulation of these two genes under folate deficiency. DOT1L activity affects cell cycle progression in various organs. Therefore, we hypothesized that DOT1L and H3K79 methylation might be of relevance to NTDs. We found that the level of DOT1L was significantly decreased in NTDs mouse brain and spine (Fig. 6B). The mRNA level results also showed that DOT1L was significantly decreased in low-folate induced NTDs fetuses (Fig. 1A and C). We demonstrate here that DOT1L-mediated H3K79me2 modification may be necessary for the epigenetic signature of NTDs and we highlight the role of H3K79 and DOT1L methylation in the signature of NTDs.

In summary, we demonstrated that H3K79me2 plays a pivotal role in downregulation of *Shh*, *Sufu* genes under folate deficiency. Dot1L is required for folate deficiency reduced H3K79me2 and downregulation of *Shh*, *Sufu* genes. Hence, H3K79me2 binding to *Shh*, *Sufu* is restored after folinic acid supplementation. Furthermore, downregulation of DOT1L and SHH-related genes was observed in neural tissues of both mouse embryos and human NTDs. In low-folate deficient human NTDs samples, we found a significant positive correlation between H3K79me2 and DOT1L by correlation analysis; it demonstrated that DOT1L-mediated H3K79me2 functions in the early embryonic neurodevelopment through interactions with the SHH pathway-related genes.

### Supplementary Information

**Additional file 1: Table S1.** ChIP-qPCR and qPCR primers.

**Additional file 2: Table S2.** Human normal andNTD samples.

**Additional file 3: Table S3.** ChIP-seq assay of association analysis.

**Additional file 4: Table S4. **ChIP-seq assay of GO.

**Additional file 5: Table S5.** ChIP-seq assay of KEGG.

## Data Availability

Datasets used in this study have been deposited in Gene Expression Omnibus (GEO) database and can be accessed through https://www.ncbi.nlm.nih.gov/geo/info/linking.html. with GEO series record GSE238081.

## References

[1] Greene ND, Copp AJ (2014). Neural tube defects. Annu Rev Neurosci.

[2] Wilde J, Petersen JR, Niswander L (2014). Genetic, epigenetic, and environmental contributions to neural tube closure. Annu Rev Genet.

[3] Seller MJ (1995). Sex, neural tube defects, and multisite closure of the human neural tube. Am J Med Genet.

[4] Mitchell LE (2005). Epidemiology of neural tube defects. Am J Med Genet C Semin Med Genet.

[5] De Wals P (2007). Reduction in neural-tube defects after folic acid fortification in Canada. N Engl J Med.

[6] Marean A, Graf A, Zhang Y, Niswander L (2011). Folic acid supplementation can adversely affect murine neural tube closure and embryonic survival. Hum Mol Genet.

[7] Stover PJ (2009). One-carbon metabolism-genome interactions in folate-associated pathologies. J Nutr.

[8] Beaudin AE, Abarinov EV, Noden DM, Perry CA, Chu S, Stabler SP (2011). Shmt1 and de novo thymidylate biosynthesis underlie folate-responsive neural tube defects in mice. Am J Clin Nutr.

[9] Fleming A, Copp AJ (1998). Embryonic folate metabolism and mouse neural tube defects. Science.

[10] Dunlevy LP, Chitty LS, Burren KA, Doudney K, Stojilkovic-Mikic T, Stanier P (2007). Abnormal folate metabolism in foetuses affected by neural tube defects. Brain.

[11] Copp AJ, Greene ND (2010). Genetics and development of neural tube defects. J Pathol.

[12] Copp AJ, Brook FA, Estibeiro JP, Shum AS, Cockroft DL (1990). The embryonic development of mammalian neural tube defects. Prog Neurobiol.

[13] Mitchell LE (2005). Epidemiology of neural tube defects. Am J Med Genet C Semin Med Genet.

[14] Peters AH, Kubicek S, Mechtler K (2003). Partitioning and plasticity of repressive histone methylation states in mammalian chromatin. Mol Cell.

[15] Cheng X, Blumenthal RM (2010). Coordinated chromatin control: structural and functional linkage of DNA and histone methylation. Biochemistry.

[16] Xie Q, Li C, Song X, Wu L, Jiang Q, Qiu Z (2017). Folate deficiency facilitates recruitment of upstream binding factor to hot spots of DNA double strand breaks of rRNA genes and promotes its transcription. Nucleic Acids Res.

[17] Zhang Q, Xue P (2013). Histone modification mapping in human brain reveals aberrant expression of histone H3 lysine 79 dimethylation in neural tube defects. Neurobiol Dis.

[18] Zhang Q, Xue P, Li H (2013). Histone modification mapping in human brain reveals aberrant expression of histone H3 lysine 79 dimethylation in neural tube defects. Neurobiol Dis.

[19] van Leeuwen F, Gafken PR, Gottschling DE (2002). Dot1p modulates silencing in yeast by methylation of the nucleosome core. Cell.

[20] Nguyen AT,  Zhang.  Y (2001). The diverse functions of Dot1 and H3K79 methylation. Genes Dev.

[21] Park G (2010). Characterization of the DOT1L network implications of diverse. Protein J.

[22]  Cattaneo P,  Kunderfranco P (2016). DOT1L-mediated H3K79me2 modification critically regulates gene expression during cardiomyocyte differentiation. Cell Death Diff.

[23] Bovio PP, Franz H, Heidrich S, Rauleac T, Kilpert F, Manke T (2019). Differential methylation of H3K79 reveals DOT1L target genes and function in the cerebellum in vivo. Mol Neurobiol.

[24] Büttner N, Johnsen SA, Kügler S, Vogel T (2010). Af9/Mllt3 interferes with Tbr1 expression through epigenetic modification of histone H3K79 during development of the cerebral cortex. Proc Natl Acad Sci U S A.

[25] Franz H, Villarreal A, Heidrich S, Videm P, Kilpert F, Mestres I (2019). DOT1L promotes progenitor proliferation and primes neuronal layer identity in the developing cerebral cortex. Nucleic Acids Res.

[26] Roidl D, Hellbach N, Bovio PP, Villarreal A, Heidrich S, Nestel S (2016). DOT1L activity promotes proliferation and protects cortical neural stem cells from activation of ATF4-DDIT3-mediated ER stress in vitro. Stem Cells.

[27] Greene ND, Stanier P, Copp AJ (2009). Genetics of human neural tube defects. Hum Mol Genet.

[28] Chen CP (2007). Chromosomal abnormalities associated with neural tube defects (II): partial aneuploidy. Taiwan J Obstet Gynecol.

[29] Chen CP (2007). Chromosomal abnormalities associated with neural tube defects (I): full aneuploidy. Taiwan J Obstet Gynecol.

[30] Lai K, Kaspar BK, Gage FH, Schaffer DV (2009). Sonic hedgehog regulates adult neural progenitor proliferation in vitro and in vivo. Nat Neurosci.

[31] Odent S, Atti-Bitach T (1999). Expression of the Sonic hedgehog (SHH)gene during early human development and phenotypic expression of new mutations causing holoprosencephaly. Hum Mol Genet.

[32] Chiang C, Litingtung Y, Lee E (1996). Cyclopia and defective axial patterning in mice lacking Sonic hedgehog gene function. Nature.

[33] Svard J, Heby-Henricson K, Persson-Lek M (2006). Genetic elimination of Suppressor of fused reveals an essential repressor function in the mammalian Hedgehog signalling pathway. Dev Cell.

[34] Stanley E, Biben C (1998). DAN is a secreted glycoprotein related to *Xenopus cerberus*. Mech Dev.

[35] Padmanabhan S, Tripathi DN (2009). Methotrexate-induced cytotoxicity and genotoxicity in germ cells of mice: intervention of folic and folinic acid. Mut Res..

[36] Pei P, Cheng X (2019). Folate deficiency induced H2A ubiquitination to lead to downregulated expression of genes involved in neural tube defects. Epigenetics Chromatin.

[37] Avagliano L, Massa V (2019). Overview on Neural tube defects: from development to physical characteristics. Birth Defects Res.

[38] MRC Vitamin Study Research Group (1991). Prevention of neural tube defects: results of the Medical Research Council Vitamin Study. Lancet.

[39] Beaudin AE, Stover PJ (2007). Folate-mediated one-carbon metabolism and neural tube defects: balancing genome synthesis and gene expression. Birth Defects Res C Embryo Today.

[40] Ichi S, Costa FF, Bischof JM (2010). Folic acid remodels chromatin on Hes1 and Neurog2 promoters during caudal neural tube development. J Biol Chem.

[41] Zhao T, Gan Q, Stokes A (2014). beta-catenin regulates Pax3 and Cdx2 for caudal neural tube closure and elongation. Development.

[42] Nguyen AT, Zhang Y (2011). The diverse functions of Dot1 and H3K79 methylation. Genes Dev.

